# Effects of non-invasive brain stimulation on spontaneous neural activity in post-stroke aphasia: a protocol for a coordinate-based meta-analysis of fALFF studies

**DOI:** 10.3389/fneur.2026.1820631

**Published:** 2026-04-22

**Authors:** Yao Rao, Jingjing Zhang, Jiawei Ni, Ming Zeng, Jiali Wu, Hui Xu, Zhiwei Zhao, Cong Wang, Chunlei Shan

**Affiliations:** 1 Department of Rehabilitation Medicine, Tongren Hospital, Shanghai Jiao Tong University School of Medicine, Shanghai, China; 2 Yuanshen Rehabilitation Institute, Shanghai Jiao Tong University School of Medicine, Shanghai, China; 3 National Research Centre for Language and Well-being, Shanghai Jiao Tong University, Shanghai, China

**Keywords:** brain activity, fALFF, non-invasive brain stimulation, post-stroke aphasia, resting-state fMRI

## Abstract

**Introduction:**

Post-stroke aphasia (PSA) is a common and debilitating sequela of stroke that severely impairs quality of life. Non-invasive brain stimulation (NIBS), particularly repetitive transcranial magnetic stimulation (rTMS) and transcranial direct current stimulation (tDCS), has increasingly emerged as a promising adjunctive approach for language recovery in patients with PSA. Although accumulating evidence supports the beneficial effects of NIBS on language outcomes, the neural mechanisms underlying these clinical improvements remain incompletely elucidated. Previous meta-analyses in PSA and stroke have synthesized multiple resting-state functional magnetic resonance imaging (rs-fMRI) indices, but no meta-analysis has specifically examined treatment-related fractional amplitude of low-frequency fluctuations (fALFF) changes after NIBS in PSA. This protocol describes a coordinate-based meta-analysis designed to quantitatively synthesize rs-fMRI data, aiming to investigate how NIBS modulates intrinsic neural activity—indexed by fALFF—in patients with PSA.

**Methods and analysis:**

Two independent reviewers will conduct a systematic search of the PubMed, Web of Science, Embase, and Cochrane databases for studies published from database inception to December 1, 2025. Eligible studies evaluating the effects of NIBS on fALFF in patients with PSA will be selected based on pre-defined criteria. Only whole-brain voxel-wise fALFF studies reporting stereotactic peak coordinates in standard space will be entered into the primary quantitative AES-SDM analysis; ROI-only studies or studies without usable coordinates will be retained for narrative synthesis. Any discrepancies arising during study selection or data extraction will be resolved through consultation with a third independent reviewer. Neuroimaging reporting quality will be assessed with a customized 20-point checklist, and risk of bias will be evaluated with design-specific tools (RoB 2 for randomized trials and ROBINS-I for non-randomized intervention studies). We will also record studies using other rs-fMRI indices (e.g., ALFF, ReHo, and functional connectivity) during screening to describe the broader evidence base. The primary outcome measures will focus on alterations in specific intrinsic regional neuronal activity assessed via rs-fMRI. The meta-analysis of neuroimaging data will be conducted using Anisotropic Effect Size Seed-Based d Mapping (AES-SDM, version 5.15), while clinical outcome analyses will be performed using RevMan 5.4 software (The Cochrane Collaboration). The reporting of this study will strictly adhere to the PRISMA (Preferred Reporting Items for Systematic Reviews and Meta-Analyses) guidelines.

**Conclusion:**

This study will quantitatively synthesize findings from independent neuroimaging studies to provide comprehensive evidence for identifying the modulation patterns of intrinsic brain activity induced by NIBS in patients with PSA.

**Systematic review registration:**

Identifier CRD420251275236. https://www.crd.york.ac.uk/PROSPERO/view/CRD420251275236

## 1 Introduction

Stroke stands as one of the leading global causes of long-term disability and mortality, with its incidence exhibiting a sustained upward trajectory (1). Clinically, approximately one-third of stroke survivors are afflicted with post-stroke aphasia (PSA) (2), a devastating sequela of stroke that impairs core language functions, including both comprehension and expression, and consequently induces severe communication barriers (3). Beyond linguistic deficits, PSA profoundly hinders patients’ performance of activities of daily living and elevates their risk of social isolation and depressive disorders (4). Although partial spontaneous language recovery may occur during the acute post-stroke phase, a substantial proportion of patients continue to experience chronic language impairments. These residual deficits impose an enormous medical, economic, and social burden on affected individuals, their families, and the broader healthcare system (5).

Speech language therapy (SLT) remains the primary treatment for aphasia (6). However, this behaviorally oriented approach has encountered significant challenges due to its variable therapeutic efficacy and the inability to directly modulate cortical excitability (7). Clinical evidence suggests that outcomes based solely on behavioral training often exhibit significant individual variability. Furthermore, a large proportion of patients reach a recovery plateau, where further functional improvement is difficult to achieve (8). To address this therapeutic bottleneck, adjunctive interventions aimed at direct modulation of brain function and enhancement of neuroplasticity have emerged as a major focus of contemporary research in rehabilitation medicine (9, 10).

Non-invasive brain stimulation (NIBS), which mainly includes repetitive transcranial magnetic stimulation (rTMS) and transcranial direct current stimulation (tDCS), is widely used in the rehabilitation treatment of PSA due to its non-invasive, painless, and easy-to-administer properties (11, 12). Most NIBS studies in aphasia have been grounded in the hypothesis of interhemispheric inhibitory imbalance, which posits that language recovery can be facilitated by either enhancing activity in the lesioned left hemisphere or suppressing maladaptive hyperactivity in the contralesional right hemisphere (13). Although NIBS has shown preliminary clinical efficacy, its underlying neurophysiological mechanisms remain incompletely understood, and existing theoretical models fail to fully explain the range of observed findings.

Resting-state functional magnetic resonance imaging (rs-fMRI) is a non-invasive technique capable of characterizing intrinsic brain functional activity and provides a valuable tool for investigating the neural mechanisms (14). Among rs-fMRI metrics, fractional amplitude of low-frequency fluctuations (fALFF) serves as a key biomarker reflecting the intensity of local spontaneous neuronal activity (15). Importantly, the existing meta-analytic literature has already shown that rs-fMRI metrics can be synthesized as observational indicators in stroke-related disorders and PSA, including meta-analyses combining amplitude of low-frequency fluctuations (ALFF), fALFF, and regional homogeneity (ReHo) to delineate common patterns of spontaneous regional dysfunction (16), as well as reviews emphasizing the role of resting-state functional connectivity (FC) in language-network reorganization (17, 18). However, these prior syntheses mainly addressed disease-related abnormalities or mixed rs-fMRI indicators rather than treatment-induced neural changes following NIBS. Despite a growing number of fALFF-based studies investigating the neural effects of NIBS in PSA, inconsistencies in sample characteristics, stimulation parameters, and disease chronicity have resulted in substantially heterogeneous findings across studies (19, 20). Specifically, while some reports highlight enhanced activation of the ipsilesional inferior frontal gyrus following NIBS (21, 22), others emphasize the suppression of maladaptive hyperactivity in contralesional homologous areas (23–25), making it difficult to draw consistent conclusions at present.

Given these inconsistencies across studies, a systematic quantitative synthesis is required. This protocol proposes a coordinate-based meta-analysis using Anisotropic Effect-Size Seed-based d Mapping (AES-SDM) to identify convergent patterns of fALFF alterations induced by NIBS in patients with PSA. AES-SDM preserves both the magnitude and direction of reported effects and enables voxel-wise assessment of heterogeneity, making it particularly suitable for neuroimaging meta-analyses. By focusing specifically on treatment-related fALFF changes and prespecifying contrast selection and coordinate handling, this protocol aims to improve methodological transparency and interpretability.

### 1.1 Objective

In this meta-analysis, we will comprehensively evaluate previous studies to assess the impact of NIBS therapy on intrinsic brain functional activity as measured by fALFF in adults with PSA. We selected fALFF *a priori* because it quantifies regional spontaneous neural activity, is less affected by nonspecific physiological noise than ALFF, and is the most consistently reported local rs-fMRI metric in intervention studies that provide coordinate data suitable for AES-SDM. Other rs-fMRI markers, such as ALFF, ReHo, and functional connectivity, are important but reflect partly different constructs and analytic assumptions; pooling them in a single coordinate-based model would increase methodological heterogeneity. Accordingly, the primary quantitative synthesis will focus on fALFF and on the most established NIBS modalities in PSA, especially rTMS/TBS and tDCS, while other modalities and non-fALFF studies will be documented for narrative synthesis or future evidence mapping.

## 2 Methods and analysis

The meta-analysis protocol has been registered with the international PROSPERO under the registration number CRD420251275236 and was developed in accordance with Preferred Reporting Items for Systematic review and Meta-Analysis Protocols (PRISMA-P) (26) (Supplementary Data 1).

### 2.1 Eligibility criteria

The inclusion and exclusion criteria for study selection are defined as follows.

### 2.2 Study types

Randomized controlled trials and longitudinal within-subject studies reported in English will be eligible, provided they investigate the effects of NIBS using resting-state fMRI with a focus on the fALFF measure. For the primary quantitative meta-analysis, only randomized or sham-controlled intervention datasets contributing an eligible between-group contrast will be pooled; non-randomized longitudinal within-subject studies and single-arm pre-post studies will be extracted separately and treated as exploratory evidence. Grey literature (e.g., conference proceedings, theses) will be included only if complete stereotactic peak coordinates (x, y, z) are available or can be obtained from the authors. Systematic reviews, meta-analyses, letters, case reports, cross-sectional studies and case series with a sample size of less than 5 will be excluded. When overlapping cohorts or duplicate data are suspected, we will retain the most informative non-duplicated dataset and use companion reports solely to supplement descriptive information. All appropriate studies published before December 1, 2025 will be included.

### 2.3 Participants

Adult patients with a clinical diagnosis of PSA confirmed by structural neuroimaging (CT or MRI) and standardized language assessments such as Western Aphasia Battery (WAB) or Boston Diagnostic Aphasia Examination (BDAE), will be included. Studies enrolling participants with either ischemic or hemorrhagic stroke, at any stage of recovery (acute, subacute, or chronic), and affecting either hemisphere will be considered. Mixed studies in which participants received concurrent conventional SLT alongside the brain stimulation intervention will be included. Studies in which participants receive concurrent conventional speech-language therapy (SLT) will also be eligible. To reduce confounding, such studies will contribute to the primary quantitative synthesis only when the background SLT program is matched or sufficiently comparable across study arms; otherwise, they will be retained for narrative or exploratory synthesis only.

Patients with aphasia resulting from non-stroke etiologies (e.g., traumatic brain injury, brain tumors, or neurodegenerative conditions such as primary progressive aphasia) or those with severe comorbid neuropsychiatric disorders that could independently alter spontaneous neural activity will be excluded. No restrictions will be applied regarding race, sex, or handedness, provided participants are adults aged 18 years or older.

### 2.4 Interventions

We will include all studies utilizing NIBS applied to the human brain to modulate neural activity. Eligible modalities include transcranial magnetic stimulation (TMS) [e.g., low- or high-frequency rTMS and theta burst stimulation (TBS)], transcranial electrical stimulation (tES) [including tDCS, transcranial alternating current stimulation (tACS), and transcranial random noise stimulation (tRNS)], and transcranial ultrasound stimulation (tUS) [e.g., transcranial focused ultrasound (FUS)]. Because the likely evidence base for fALFF studies is small, rTMS/TBS and tDCS will be regarded as the core modalities for primary pooled analyses whenever feasible, whereas less common modalities (e.g., tACS, tRNS, tUS/tFUS) will be summarized descriptively and considered for exploratory subgroup analyses only if at least three independent datasets are available.

Both single-session studies investigating immediate neurophysiological effects and repeated-session studies examining longitudinal therapeutic outcomes will be included. Interventions may be administered as standalone treatments or as adjuvant therapies combined with conventional SLT, provided that the stimulation itself is the primary variable of interest. Studies employing invasive stimulation methods (e.g., deep brain stimulation), high-intensity focused ultrasound (HIFU) used for thermal ablation, or electroconvulsive therapy (ECT) will be excluded.

### 2.5 Comparators

Eligible comparators mainly consist of sham stimulation control groups, where participants receive sham procedures—such as sham TMS coils or inactive tDCS/tUS protocols—alongside comparable concomitant care like conventional SLT. Randomized or sham-controlled comparisons with matched concomitant therapy will constitute the primary inferential dataset. Longitudinal within-subject comparisons (post-treatment versus pre-treatment rs-fMRI within the active NIBS group) will also be collected, but these contrasts will be analyzed separately as exploratory evidence rather than pooled with between-group effects. However, studies that only compare patients with healthy controls (which reflect disease pathology rather than treatment effects) or compare different active interventions without a sham or pre-treatment baseline reference will be excluded.

### 2.6 Outcome measures

The primary outcome will be alterations in intrinsic regional neuronal activity assessed using rs-fMRI with fALFF as an outcome measure. This restriction is prespecified to maximize construct homogeneity across studies. Only whole-brain voxel-wise analyses reporting significant stereotactic peak coordinates (x, y, z) in standard MNI or Talairach space will be eligible for quantitative AES-SDM synthesis; studies reporting only region-of-interest analyses, graph-theory summaries, or qualitative statements without usable coordinates will be retained for narrative synthesis. Talairach coordinates will be converted to MNI space before analysis. When multiple statistical outputs are available for the same dataset, we will prioritize corrected whole-brain results. A hierarchical extraction strategy will be applied: group-by-time interactions from randomized trials first, post-treatment between-group differences second, and within-group pre-post contrasts only for exploratory analyses. Between-group and within-group contrasts will be analyzed separately. The primary time point for analysis will be immediately post-intervention, with available long-term follow-up data also being documented.

Secondary outcomes will include: (i) evaluation of clinical efficacy as reflected by changes in standardized language assessment scores (e.g., the WAB or BDAE); (ii) investigation of brain–behavior associations by correlating NIBS-induced fALFF alterations with clinical language improvements; and (iii) assessment of safety by monitoring the incidence of adverse events.

### 2.7 Searching strategy

Two independent reviewers will conduct a comprehensive systematic search across major electronic databases, including PubMed, Embase, Web of Science, and the Cochrane Library, from their inception up to December 1, 2025. The search strategy will employ a combination of Medical Subject Headings (MeSH) and relevant keywords. Boolean operators (AND/OR) will be used to combine these terms. To improve specificity, the search strategy will explicitly pair aphasia-related terms with stroke-related terms (e.g., “post-stroke aphasia,” “stroke aphasia,” “cerebrovascular accident” AND aphasia), restrict imaging terms to resting-state functional MRI terminology (e.g., “resting-state fMRI,” “rs-fMRI,” “functional magnetic resonance imaging”, “BOLD”) rather than generic MRI terms, and include fALFF-specific keywords (e.g., “fractional amplitude of low-frequency fluctuation” OR “fALFF”). During full-text screening, we will record the number of studies identified for other rs-fMRI metrics and NIBS modalities, and we will use these data to transparently reassess whether a broader future synthesis across rs-fMRI metrics would be feasible. The Pubmed database-tailored search strings are provided in Supplementary Data 2.

## 3 Data collection and analysis

### 3.1 Selection process

**Figure 1:**
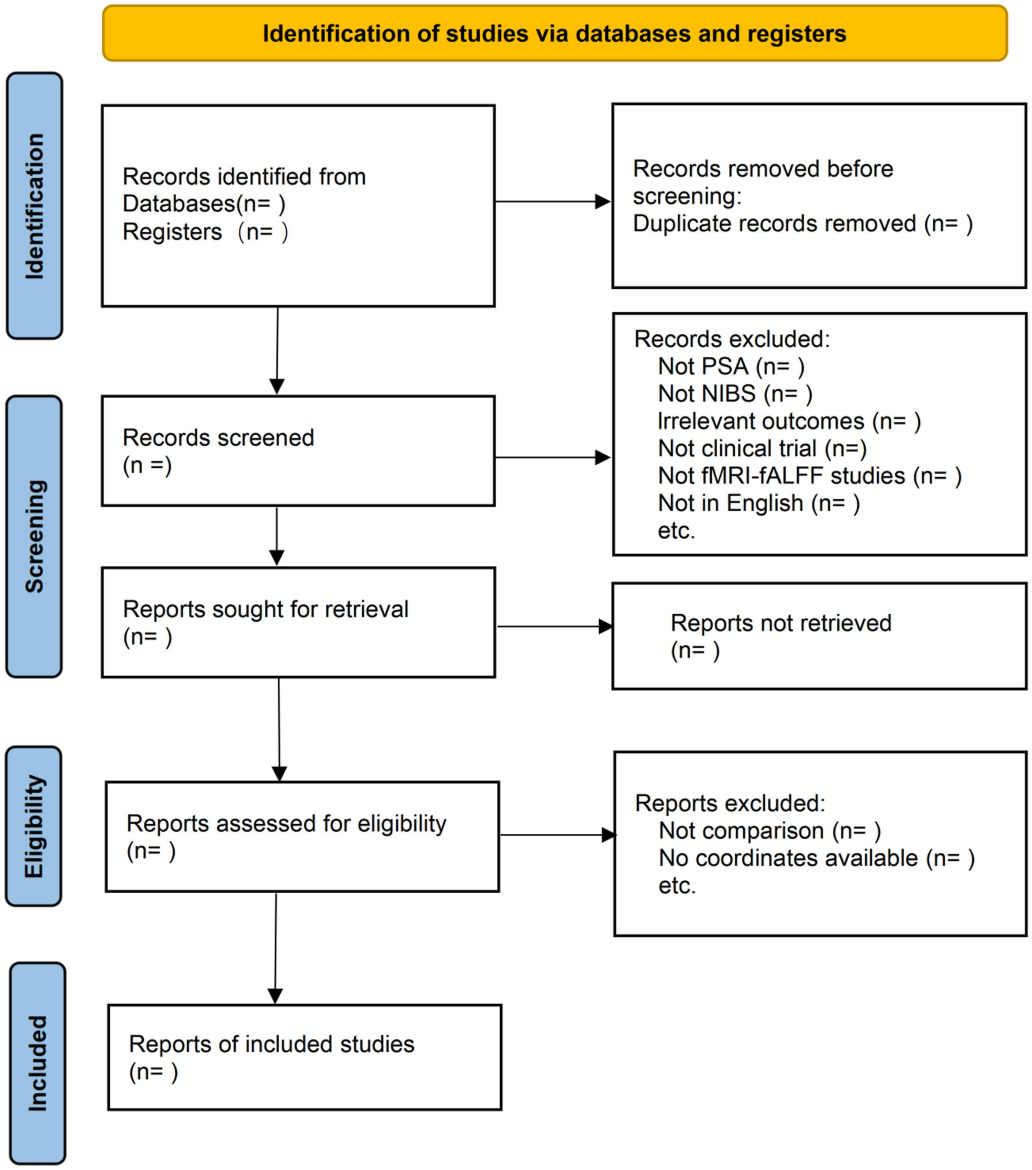
PRISMA flow diagram of study-selection process. PSA, post-stroke aphasia; NIBS, non-invasive brain stimulation; fMRI, functional magnetic resonance imaging; fALFF, fractional amplitude of low-frequency fluctuations.

Reference management and deduplication will be performed using EndNote X9 software (Thomson Reuters, New York, USA). Following the removal of duplicates, two reviewers will independently screen the titles and abstracts to exclude clearly irrelevant records. Potentially eligible studies will then undergo a comprehensive full-text review to determine final inclusion based on the pre-defined criteria. Any discrepancies arising during the screening process will be resolved through discussion or consultation with a third independent reviewer to reach a consensus. The entire selection process will be rigorously documented and presented in a PRISMA (27) flow diagram (Figure 1) to ensure methodological transparency and reproducibility.

### 3.2 Data collection

Data extraction will be performed independently by two reviewers using a standardized, pre-piloted data extraction form. Any discrepancies will be resolved through discussion, with a third independent reviewer acting as an arbiter for final adjudication if necessary. The extraction form will explicitly document all eligible contrasts within a study, the statistical threshold used (corrected or uncorrected), coordinate space, effect direction, and whether results derive from randomized comparisons or within-group change analyses, so that potentially conflicting findings can be handled transparently according to the prespecified prioritization rules. The following data will be systematically extracted:

1. Study characteristics: bibliographic information (first author, year, country), study design (e.g., RCT, longitudinal pre-post intervention), and sample size.
2. Participant profile: mean age, gender distribution, handedness, time post-stroke, aphasia type and severity.
3. Intervention protocols: NIBS modality (rTMS/tDCS/tUS), target site, stimulation parameters (frequency, intensity, duration), total number of sessions, and details of concomitant speech therapy.
4. Comparator details: specifics of the control condition (e.g., sham TMS coil type, electrode placement for sham tDCS) or pre-intervention baseline characteristics.
5. Outcome measures: stereotactic peak coordinates (x, y, z) of statistically significant fALFF changes, coordinate space, direction of change, statistical details. When studies report multiple relevant outputs, we will record all potentially eligible results but select only one non-duplicated contrast per dataset for the primary analysis according to the predefined hierarchy (interaction > post-treatment between-group > within-group pre-post). In addition, during screening we will tabulate studies that meet the clinical/intervention criteria but report ALFF, ReHo, or functional connectivity instead of fALFF, so that the scope of the broader rs-fMRI evidence base can be described transparently.

The extracted data will be summarized in a ‘Characteristics of Included Studies’ table, and reasons for any full-text exclusions will be documented in detail.

### 3.3 Data synthesis

Extracted data will be systematically tabulated to provide a comprehensive overview of the included studies. The final summary table will explicitly record the following variables for each study: bibliographic information (first author, publication year), study design, sample characteristics (sample size per group, mean age, gender ratio, handedness, stroke chronicity, aphasia type), intervention protocols (NIBS modality, stimulation target site, frequency and intensity, session duration, total number of sessions, concurrent speech therapy), control conditions, neuroimaging acquisition details (scanner strength, TR/TE), and clinical outcome measures. For the neuroimaging data, a quantitative, random-effects coordinate-based meta-analysis will be conducted using the AES-SDM method. AES-SDM was selected over alternative coordinate-based approaches because it preserves both the magnitude and direction of effects, allows incorporation of null findings, and enables voxel-wise heterogeneity and meta-regression analyses. This method enables the identification of spatially convergent patterns of neural activity alterations following NIBS intervention. The primary pooled analysis will be limited to randomized or sham-controlled whole-brain voxel-wise fALFF datasets, with exploratory analyses conducted separately for within-subject longitudinal evidence and for less common stimulation modalities only when the number of datasets is sufficient. If the refined search yields too few eligible fALFF datasets for robust quantitative synthesis, we will explicitly report this feasibility constraint and retain the review as a systematic and narrative summary rather than forcing an underpowered meta-analysis.

### 3.4 Quality assessment

To evaluate the methodological quality of the included neuroimaging studies, we will employ a customized 20-point checklist adapted from previous peer-reviewed meta-analyses (28, 29). This checklist is intended to assess the completeness and transparency of neuroimaging reporting rather than to serve as a stand-alone risk-of-bias tool. Therefore, we will complement it with design-specific bias assessment using RoB 2 (30) for randomized controlled trials and ROBINS-I (31) for non-randomized intervention studies. We will extract key information from each study, including the first author, year of publication, sample size, gender distribution, age, scanner field strength, and statistical threshold. To prepare for the subsequent voxel-wise meta-analysis, peak cluster coordinates and effect sizes (e.g., *t*-values or *z*-scores) will be recorded. The checklist is shown in Table 1.

**Table 1:** Quality assessment of individual studies: criteria for objective methodological evaluation.

**Category**	**Criteria**	**Score**
**Category 1: Sample characteristics (10)**	Patients were evaluated with specific standardized diagnostic criteria	1
	Important demographic data (age and gender) were reported with mean (or median) and standard deviations (or range))	2
	Healthy comparison subjects were evaluated to exclude psychiatric and medical illnesses and demographic data was reported	1
	Important clinical variables (e.g. illness duration, onset time, medication status, WAB or BDAE scores) were reported with mean (or median) and standard deviations (or range)	4
	Sample size per group > 10	2
**Category 2: Methodology and reporting (10)**	Whole brain analysis was automated with no a-priori regional selection	3
	Magnet strength at least 1.5T	1
	At least 5 minutes of resting state acquisition	1
	Whole brain coverage of resting scans	1
	The acquisition and preprocessing techniques were clearly described so that they could be reproduced	1
	Coordinates reported in a standard space	1
	Significant results are reported after correction for multiple testing using a standard statistical procedure (FDR, FWE or permutation-based methods)	1
	Conclusions were consistent with the results obtained and the limitations were discussed	1

WAB, Western Aphasia Battery; BDAE, Boston Diagnostic Aphasia Examination; FDR, false discovery rate; FWE, family-wise error.

### 3.5 Estimation of the treatment effect

Voxel-wise coordinate-based meta-analysis will be conducted using the AES-SDM software package (32). Specifically, we will first extract stereotactic peak coordinates of clusters showing significant differences from each included study, which will then be used to reconstruct effect-size maps of group differences employing an anisotropic unnormalized Gaussian kernel. Subsequently, these individual study maps will be combined into a mean meta-analytic map via a random-effects model, wherein each study is weighted by the inverse of the sum of its within-study variance and between-study heterogeneity (33). To identify significant regional alterations, we will apply the standard default AES-SDM thresholding parameters: a kernel size of FWHM = 20 mm, a voxel-level uncorrected probability of *p* < 0.005, a peak height threshold of SDM-Z > 1.0, and a minimum cluster extent of > 10 voxels, with all results visualized on a standard anatomical template in MNI space.

Clinical outcomes from randomized comparisons and single-arm pre-post studies will be synthesized separately because their effect-size structures differ. For continuous clinical variables (e.g., changes in language scores), we will calculate the mean difference (MD) with a 95% confidence interval (CI) when studies utilize identical scales. If different scales are used (e.g., WAB vs. BDAE), the standardized mean difference (SMD) with a 95% CI will be employed. Results will be visualized through forest plots. Publication-bias analyses such as funnel plots and Egger’s test will be considered only when a sufficient number of independent datasets contribute to a given contrast or cluster (preferably ≥10); otherwise, they will be omitted or interpreted as exploratory.

Given the expected small number of eligible fALFF studies, some planned subgroup, meta-regression, and publication-bias analyses may be conducted only if sufficient datasets are available and should be interpreted as exploratory.

### 3.6 Sensitivity analysis

We will perform a jackknife sensitivity analysis to examine the robustness of the neuroimaging findings. In this leave-one-out procedure, the meta-analysis will be repeated multiple times, each time omitting one dataset. A brain region will be considered relatively robust when it remains significant across most jackknife iterations (33). Sensitivity analyses will be undertaken only when the number and design of available datasets make such testing meaningful; therefore, not every planned sensitivity procedure will necessarily be applicable to every outcome or subgroup.

### 3.7 Heterogeneity analysis

Heterogeneity across studies will be assessed using a random-effects model. This model incorporates the Q statistic. Voxel-wise heterogeneity maps are calculated to detect brain regions with significant variability in effect sizes. Voxel-wise heterogeneity will be examined using Q statistics as implemented in AES-SDM, and heterogeneity summaries (including *I*^2^ where applicable) will be interpreted descriptively. Values of 25, 50, and 75% indicate low, moderate, and high heterogeneity, respectively. Significant heterogeneity (defined as *p* < 0.05) requires cautious interpretation of the brain clusters. Consequently, we will investigate these regions further using subgroup or meta-regression analyses.

### 3.8 Subgroup analysis

Significant heterogeneity, defined as *p* < 0.05 for the *Q* statistic, warrants cautious interpretation of the identified brain clusters. To address this issue, we will perform pre-planned subgroup analyses to explore potential sources of variability. Specifically, studies will be stratified according to stimulation modality (e.g., low-frequency rTMS, high-frequency rTMS, and anodal tDCS), stimulation site (e.g., left- versus right-hemisphere targets), and stroke chronicity (e.g., subacute versus chronic phase), provided that a sufficient number of datasets (*N* ≥ 3 per subgroup) are available. Less common modalities such as tACS, tRNS, and tUS/tFUS will not be pooled indiscriminately with established modalities unless enough homogeneous datasets are available to support an interpretable exploratory subgroup.

### 3.9 Meta-regression analysis

To further investigate the association between neural alterations and clinical characteristics, voxel-wise meta-regression analyses will be conducted using the AES-SDM software. Specifically, we will examine the relationship between fALFF effect sizes and continuous variables, including clinical language improvement (indexed by changes in WAB-AQ or BDAE scores) as well as demographic and clinical factors (e.g., mean age and time since stroke). Meta-regression analyses will be performed only when data for the relevant covariates are available in at least 10 independent studies, in order to ensure sufficient statistical power and reduce the risk of spurious findings.

### 3.10 Narrative synthesis

For eligible studies that meet the inclusion criteria but are not suitable for the quantitative coordinate-based meta-analysis (e.g., studies that report only Region-of-Interest analyses without stereotactic coordinates or those with missing data that authors were unable to provide), a narrative synthesis will be performed. Key characteristics of these studies—such as sample size, population, and intervention protocols—along with their main findings will be systematically summarized in a dedicated table. The synthesis will focus on qualitatively describing the direction of reported changes in fALFF (increase or decrease) and the corresponding anatomical regions (e.g., specific gyri, left vs. right hemisphere). Narrative synthesis will also be used for less common NIBS modalities, studies with imbalanced or poorly described concurrent SLT, and non-randomized within-subject datasets that are not eligible for the primary pooled comparison. We will compare these qualitative findings with the main AES-SDM results to assess consistency and identify possible sources of heterogeneity.

### 3.11 Publication bias

Publication bias will be assessed for brain clusters exhibiting significant fALFF alterations. This will be achieved through the visual inspection of funnel plots and the quantitative application of Egger’s regression test. The analysis will be conducted specifically for the peak coordinates of the identified significant clusters. Because such tests are unreliable with sparse datasets, they will only be applied when a sufficient number of independent studies contribute to the analysis; otherwise, we will report that publication bias could not be robustly assessed. A *p*-value < 0.05 in Egger’s test will be considered indicative of significant publication bias.

### 3.12 Confidence in cumulative evidence

Formal GRADE assessment will not be applied, as coordinate-based neuroimaging outcomes do not directly map onto intervention effect estimates; instead, confidence will be inferred from consistency, robustness, and heterogeneity of findings.

### 3.13 Patient and public involvement

Patients and the public will not be involved in the design, conduct, reporting, or dissemination of this research.

### 3.14 Ethics and dissemination

Ethical approval is not required for this study, as it is a meta-analysis based on the synthesis of data derived from previously published, peer-reviewed literature. No new human data will be collected, and there will be no direct intervention with human subjects.

## 4 Discussion

This coordinate-based meta-analysis will be the first to quantitatively synthesize rs-fMRI findings on the effects of NIBS on brain activity in PSA. We will employ the AES-SDM approach, which is particularly suitable for integrating results from small studies and addressing heterogeneity across experiments. This method allows the identification of convergent patterns of brain activity alterations, providing insights into the neurophysiological mechanisms underlying NIBS-induced language recovery. In contrast to previous meta-analyses that combined multiple rs-fMRI indicators mainly to describe disease-related abnormalities, the present protocol targets one prespecified intervention-sensitive marker to improve methodological coherence and interpretability. Precise localization of these changes is critical for distinguishing adaptive from maladaptive neuroplasticity and may inform the optimization of future stimulation targets and protocols to enhance therapeutic efficacy for PSA.

Considerable variability in NIBS parameters, lesion sites, and device characteristics is expected across included studies, which may complicate data integration. We therefore place randomized or sham-controlled comparisons at the center of the primary quantitative synthesis and reserve within-subject or less common-modality evidence for exploratory interpretation. This approach is intended to balance comprehensiveness with methodological reliability.

This study has several limitations. First, variability in stroke lesion size and location across patients may influence the observed results. Second, differences in NIBS protocols, stimulation parameters, and device characteristics may obscure the true effects of the intervention. Third, only studies published in English will be included, potentially leading to language bias. Finally, studies that do not report precise stereotactic coordinates will be excluded, which may result in the loss of some relevant data. Moreover, by focusing on fALFF we may omit informative evidence derived from ALFF, ReHo, or functional connectivity; however, we consider this trade-off justified for the current protocol because a narrower metric definition improves internal validity, and the revised screening process will still document the size and characteristics of the broader rs-fMRI literature. We also acknowledge that concurrent SLT may remain a source of residual heterogeneity even after restricting the primary synthesis to studies with matched or comparable background therapy.
